# Impact of Aspirin Eugenol Ester on Cyclooxygenase-1, Cyclooxygenase-2, C-Reactive Protein, Prothrombin and Arachidonate 5-Lipoxygenase in Healthy Rats

**Published:** 2017

**Authors:** Ning Ma, Guan-Zhou Yang, Xi-Wang Liu, Ya-Jun Yang, Isam Mohamed, Guang-Rong Liu, Jian-Yong Li

**Affiliations:** a *Key Lab of New Animal Drug Project of Gansu Province, Key Lab of Veterinary Pharmaceutical Development, Ministry of Agriculture, Lanzhou Institute of Husbandry and Pharmaceutical Science of CAAS, Lanzhou730050, P.R. China. *; b *School of Pharmacy Lanzhou University, Lanzhou 730020, P.R. China.*

**Keywords:** Aspirin eugenol ester (AEE), Chemical-protein interactions, Thrombosis, Inflammation, Rats

## Abstract

Aspirin eugenol ester (AEE) is a promising drug candidate which is used for the treatment of inflammation, pain, fever, and the prevention of cardiovascular diseases. This study focuses on the effect of AEE on five proteins which are related to inflammation and thrombosis, including cyclooxygenase-1 (COX-1), cyclooxygenase-2 (COX-2), C-reactive protein (CRP), prothrombin (FII) and arachidonate 5-lipoxygenase (ALOX5). Meanwhile, the study was administrated to compare the drug effect between AEE and its precursor from the view of chemical-protein interactions. Healthy rats were given AEE, aspirin, eugenol and integration of aspirin and eugenol. Carboxyl methyl cellulose sodium (CMC-Na) was used as control. After drugs were administered intragastrically for seven days, the blood samples were collected to measure the proteins concentration by enzyme linked immuno-sorbent assay (ELISA). The results showed that the concentrations of key endogenic bioactive enzymes were significantly reduced in AEE groups when compared with CMC-Na and aspirin groups (*P *< 0.01). Drug effects of AEE on five proteins were stronger than aspirin and eugenol. From the view of chemical-protein interactions, AEE had positive effects on anti-inflammation and anti-thrombosis and showed stronger effects than aspirin and eugenol.

## Introduction

As a classical drug, aspirin has been widely used for treatment of inflammation, headache, fever and cardiovascular diseases more than one century (1-4). The biochemical mechanism of aspirin has been described previously (5-7). Aspirin produces its therapeutic and side effect via inhibitation of cyclooxygenase (COX), which is a key enzyme to catalyze prostaglandin formation (6). Later on, aspirin was successfully extended to prevent and treat of cardiovascular diseases since the inhibition of thromboxane A2 (TXA2) which is a crucial factor for blood clotting (2). Recently, the mechanism of aspirin in anti-cancer and anti-aging also has been confirmed (8-10) .

Eugenol is the main component of volatile oil extracted from dry alabastrum of *Eugenia Caryophyllata Thumb* and used as a fragrance ingredient in many dietary products. Several pharmacological activities of eugenol have been demonstrated, including antivirus, antibacterial, antipyretic, analgesia, anti-inflammation, anti-platelet aggregation, anticoagulation, antioxidation, anti-diarrhea, anti-hypoxia and, antiulcer, and inhibition of intestinal movement and arachidonic acid metabolism (11-19).

However, the adverse reactions of aspirin such as gastrointestinal damage, have limited the long-term use of this old drug (20). Eugenol which contains phenolic hydroxyl group, is irritative and can be easily oxidized. In order to reduce these side effects and improve the efficacy, an aspirin eugenol ester (AEE) was synthesized recently (21). AEE is a colorless transparent crystal. The acute toxicity, pharmacodynamics, stability, teratogenicity and mutagenicity of AEE have been investigated. The LD_50_ of AEE is 10.39 g/kg, which is 0.02 times of aspirin and 0.27 times of eugenol (22). A 15-day oral dose toxicity study as well as a comprehensive evaluation of genotoxicity by Ames test and the mouse bone marrow micronucleus assay were carried out. The results showed that its toxicity was not significant (22, 23). Its anti-inflammatory, analgesic, and antipyretic effects were similar to aspirin and eugenol, but lasted for a longer period (24, 25). Recently, metabolites of AEE in beagle dog also have been confirmed by HPLC-MS/MS (26).

**Figure 1:**
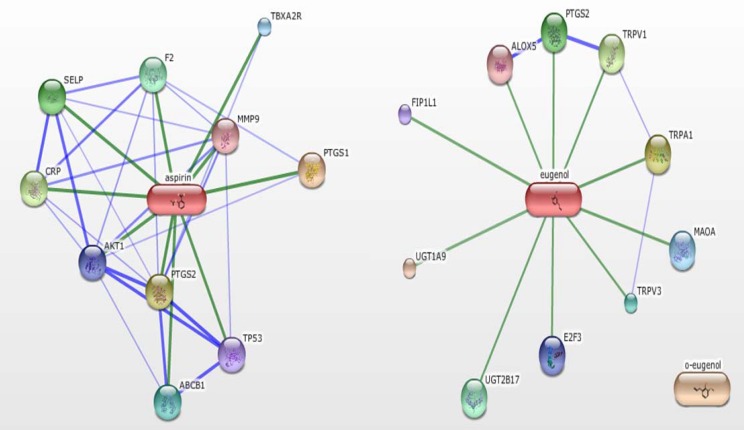
The confidence view of aspirin and eugenol from chemical-protein interactions. Through searching the database of STICH, proteins linked to aspirin and eugenol were found out. Stronger associations are represented by thicker lines. Protein-protein interactions are shown in blue, chemical-protein interactions in green. Through the network, the key substances from inflammation and thrombosis were selected. A: Aspirin as the center, B: Eugenol as the center

STITCH (Search Tool for Interactions of Chemicals), is a well-known database containing both of the known and predicted interactions of chemicals and proteins (http://stitch.embl.de/) (27). A general way to evaluate candidate drugs is to analyze the relationship between the candidate drugs and the targeted substances which are related to disease (28-30). Thus, the information detailing chemical-protein interactions of aspirin and eugenol was employed and retrieved from STITCH (Figure 1). Several proteins related to inflammation and thrombosis were chosen in the study, including COX-1, COX-2, CRP, FII, and ALOX5 (Table 1). The main idea behind this method is based on the fact that the compounds and their precursor often share similar functions. Therefore, this study focused on investigating the influence of AEE on these proteins and comparing effects between AEE and its precursor. This study will help to partly clarify the effects of AEE from protein-chemical interactions, which can provide guidance for the further study of AEE.

## Experimental

*Chemicals and reagents*

AEE; transparent crystal (purity: 99.5% with RE-HPLC), was prepared in key lab of new animal drug project of Gansu Province, key lab of veterinary pharmaceutical development of Agricultural Ministry, Lanzhou institute of Husbandry, and pharmaceutical sciences of CAAS. CMC-Na (carboxyl methyl cellulose sodium) was supplied by Tianjin Chemical Reagent Company (Tianjin, China). Aspirin and Tween-80 were supplied by Aladdin Industrial Corporation (Shanghai China). Eugenol was supplied by Sinopharm Chemical Reagent Co., Ltd. (Shanghai China). ELISA kits of COX-1, COX-2, CRP, FII and ALOX5 were purchased from U.S.A TSZ biological Trade Co., Ltd. (Lexington, USA). Multiskan Go 1510 as spectrophotometer was supplied by Thermo Fisher Scientific (USA).

**Figure 2:**
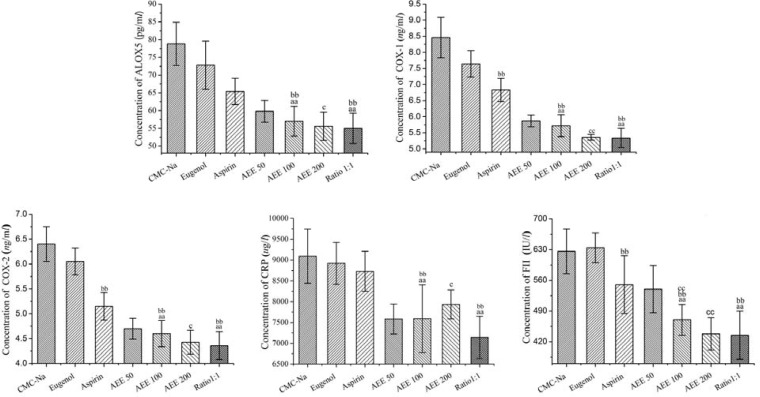
Effects of different drugs on key endogenic bioactive enzymes after drugs administration for seven days (n = 10). The concentrations of enzymes in serum were measured by ELISA method. Values were presented as mean ± SD. Multiple comparisons were carried out to find out the difference between AEE and its precursor. ^aa^*P *< 0.01 compared with aspirin group. ^bb^*P *< 0.01 compared with eugenol group. ^c^*P *< 0.05, ^cc^*P *< 0.01 compared with low-dose of AEE group. Ratio 1:1, integration of aspirin and eugenol, molar ratio 1:1, at 0.3067 mmol

**Table 1:** The basic information of key endogenic bioactive enzymes

**Substance (Abbreviation)**	**Full Name**	**PDB Number**	**Inflammation**	**Thrombosis**
ALOX5	Arachidonate 5-lipoxygenase	P09917	+	-
COX-1	Prostaglandin-endoperoxide synthase 1	P23219	+	-
COX-2	Prostaglandin-endoperoxide synthase 2	P35354	+	-
FII	Coagulation Factor II	P00734	-	+
CRP	C-reaction protein	P02741	+	-

Notes: The key endogenic bioactive enzymes including five proteins were found out in PDB. Based on the different effects in inflammation and thrombosis, the key endogenic bioactive enzymes were divided into two categories: one category has a close relationship with inflammation; another has a close relationship with thrombosis. +: close relation; -: loose relationship

**Table 2:** The experimental design in the study

**Groups**	**Drug**	**Dosage** ** (** **mg/kg** **)**	**Average volume** **per rat (mL)**
A	CMC-Na	50	1.74
B	Eugenol	50.3	1.63
C	Aspirin	55.2	1.73
D	AEE	50	1.65
E	AEE	100	1.72
F	AEE	200	1.76
G	Aspirin + eugenol	55.2 + 50.3	1.68

Note: A: CMC-Na group, B: model group, C: aspirin group, D: AEE low dose, E: AEE medium dose, F: AEE high dose, G: Ratio 1:1, integration of aspirin and eugenol, molar ratio 1:1, 0.3067 mmol. In order to compare the difference between AEE and its precursors, the mole of the doses in medium-dose of AEE, aspirin and eugenol groups are the same as 0.3067 mmol. The averaged intragastrically volume in different groups were approximate equal

**Table 3:** The actual concentrations of key endogenic bioactive enzymes in each group with different drugs

**Group**	**ALOX5 (pg/mL)**	**CRP (μg/L)**	**FII (IU/L)**	**COX-1 (ng/mL)**	**COX-2 (ng/mL)**
CMC-Na	78.8 ± 6.1	9093 ± 652	626 ± 51	8.46 ± 0.63	6.4 ± 0.35
Eugenol	72.8 ± 6.8	8921 ± 503	634 ± 34	7.64 ± 0.41**	6.05 ± 0.27*
Aspirin	65.4 ± 3.7**	8731 ± 480	550 ± 66*	6.83 ± 0.36**	5.15 ± 0.28**
Low-dose AEE	59.8 ± 3.1**	7585 ± 360**	540 ± 54**	5.87 ± 0.18**	4.7 ± 0.21**
Medium-dose AEE	57 ± 4.2**	7592 ± 815**	470 ± 35**	5.72 ± 0.34**	4.6 ± 0.26**
High-dose AEE	55.6 ± 4**	7935 ± 347**	438 ± 37**	5.36 ± 0.09**	4.43 ± 0.24**
Ratio 1:1	55 ± 4.3**	7142 ± 510**	435 ± 55**	5.34 ± 0.3**	4.36 ± 0.28**

Notes: After getting the average value of OD_450_ and regression equation, the diluted sample concentrations were got firstly, and then the actual concentrations were got by multiplying dilution factor. The results were expressed as mean ± SD. ANOVA in SPSS was used in data analysis.

* *P *< 0.05,

** *P *< 0.01. Significant difference from CMC-Na group. Ratio 1:1, integration of aspirin and eugenol, molar ratio 1:1, at 0.3067 mmol.

*Animals*

Seventy Wistar male rats with clean grade, aged 7 weeks and weighing 150~160 g, were purchased from the animal breeding facilities of Lanzhou Army General Hospital (Lanzhou, China). They were housed in plastic cages of appropriate size with stainless steel wire cover and chopped bedding. Light/dark regime was 12/12 h and living temperature is (22 ± 2) °C with relative humidity of (55 ± 10) %. Standard compressed rat feed from Beijing Keao Xieli Co., Ltd. (Beijing China) and drinking water were supplied ad libitum. The study was performed in compliance with the Guidelines for the care and use of laboratory animals as described in the US national institutes of health and approved by institutional animal care and use committee of Lanzhou institute of husbandry and pharmaceutical science of CAAS. Animals were allowed a 2-week quarantine and acclimation period prior to start of the study.

*Drug preparation*

AEE and aspirin suspension liquid were prepared in 0.5% of CMC-Na. Eugenol and Tween-80 at the mass ratio of 1:2 were mixed with the distilled water to prepare eugenol emulsion.

*Dose and sample collection*

Animal disease models have been used to evaluate the effects of AEE on anti-inflammatory, analgesic, and antipyretic. Doses of 1.1 and 0.56 mmol/kg were chosen as high and low dosage in xylene-induced ears swelling model to investigate the anti-inflammatory effects of AEE. The results showed that xylene-induced ear swelling degree was significantly inhibited by AEE administration for three days at 0.56 mmol/kg (182.56 mg/kg) (21). When the factors including the using of normal rats, administration time and dose were taken into consideration in this experiment, the high-, medium-, and low-doses of AEE were selected as 200, 100, and 50 mg/kg, and finally, seven days as administration time were designed in the experiment. For the comparability of the experimental results, the mole of the doses in medium-dose of AEE, aspirin and eugenol groups are the same as 0.3067 mmol. Based on this experimental design, the doses of aspirin and eugenol were selected as 55.2 mg/kg and 50.3 mg/kg, respectively.

In integration of aspirin and eugenol group, aspirin and eugenol at the molar ratio 1:1 (0.3067 mole) were also designed in order to compare AEE and its precursor. The 0.5% of CMC-Na at the dosage of 50 mg/kg was as the negative control and the volume of CMC-Na was close to equal in comparison with AEE and aspirin groups (seen in Table 2). The drugs were intragastrically administered in each rat based on individual daily body weights for seven days. After seven days drug administration, the rats were anesthetized with 10% chloral hydrate and blood was collected from the abdominal aorta. Blood samples were collected into vacuum tubes without anticoagulant and then centrifuged at 1000×g for 20 min at 4 °C. Serum was stored at -80 °C until the day of analysis.

*Study design*

Based on prodrug principal, aspirin and eugenol were combined into AEE. After absorption, AEE was decomposed into salicylic acid and eugenol which are the major metabolites of AEE (26). Therefore, the proteins interacted with aspirin and eugenol were found out by the SMILE string of aspirin and eugenol by searching STITCH (seen in Figure 1). Chemicals are linked to proteins by evidence derived from experiments, databases, and the literature. In these linked proteins, the substances COX-1, COX-2, CRP, FII and ALOX5, which have close relationship with inflammation and thrombosis, were selected as the research objects. COX-1, COX-2, CRP, and ALOX5 are related to inflammation, while FII is responsible for the formation of thrombosis (seen in Table 1).

*Statistical analysis*

In the experiment, the OD value was tested four times at last; averaged, then the value into equation for sample concentration analyzed by ANOVA for unpaired groups was tested too. All data are expressed as mean ± standard deviation (SD), using SPSS 19 (Statistical Product and Service Solutions). P-value below 0.05 was considered as statistical significance.

## Results

*The OD*_450_ *value and the concentration*

In the experiment, the serum was diluted 5-fold by sample dilution. In order to reduce the experimental error, the sample was tested four times with spectrophotometer under 450 nm. After getting the first-hand data, the average value of sample wells was calculated. Based on the straight line regression equation of the standard curve, the enzyme concentrations were got with the average sample OD_450_ value in the equation, and as multiplied by the dilution factor, the results were the actual concentrations (seen in Table 3).

*The effects of AEE*

When compared with CMC-Na group, the concentration of COX-1 and COX-2 were reduced significantly in eugenol group. There were also significant differences between aspirin and CMC-Na groups. The values of ALOX5, FII, COX-1 and COX-2 were reduced significantly. When compared aspirin and eugenol groups, the values of COX-1, COX-2 and FII in aspirin group significantly showed difference from the values in eugenol group (Figure 2). The results indicated that both eugenol and aspirin had an influence on COX-1 and COX-2. In addition, the influence of aspirin on COX-1, COX-2 and FII is stronger than eugenol. However, aspirin and eugenol had no effect on CRP index.

All rats in low-, medium- and high-dose AEE groups showed a significant difference compared with the CMC-Na group (*P *< 0.01, Table 3). AEE was designed at different dose in the experiment. The concentration changes of the target proteins had a certain relationship with the AEE dosage. With the increase of AEE dose, the mean concentration values of FII, ALOX5, COX-1, and COX-2 were in decline besides CRP value. The CRP concentration in AEE high-dose group was higher than the values in low and medium groups. The results in different groups were shown in Figure 2. When compared with the values in low-dose AEE group, the values in high-dose AEE group possessed significant difference. High-dose AEE produced stronger effect on the concentration of the target proteins than medium-dose AEE group. However, the difference between medium- and high-dose AEE groups was not statistically significant. 

From the results, when compared with aspirin and eugenol groups, medium-dose AEE can significantly reduce the concentration values of all selected key endogenic bioactive enzymes (*P* < 0.01). These data indicated that AEE had greater effects than aspirin and eugenol at the same molar quantity and also demonstrated that there was a significant difference between AEE and its precursor. Meanwhile, there was no statistical difference between medium-dose AEE group and Ration 1:1 group (integration of aspirin and eugenol group, molar ratio 1:1, equal molar quantity with medium-dose AEE).

## Discussion

Felix Hoffman synthesized acetylsalicylic acid in 1897, which was the first nonsteroidal anti-inflammatory drug (NSAID) (31). Nowadays, NSAIDs are among the most commonly used drugs worldwide and their analgesic, anti-inflammatory and anti-pyretic therapeutic properties are thoroughly accepted. More than 30 million people use NSAIDs every day, and they account for 60% of the US over-the-counter analgesic market (32). AEE is being developed for anti-inflammatory, analgesic, antipyretic, anti-atherosclerosis and anti-thrombosis pharmaceutical. Metabolites of AEE *in-vivo* and *in-vitro* have been confirmed by HPLC-MS/MS (26). Five metabolites of AEE were detected in the experimental condition, including salicylic acid, salicylic acid glucuronide, salicylic acid glycine, eugenol glucuronide and eugenol sulfate. Previous studies showed that the effect of AEE is similar to aspirin and eugenol but last for long time, which indicated that AEE could be a promising NSAID candidate (21). Due to the significant efficacy against inflammation, the action mechanism of AEE is necessary to be understood. In this study, STITCH database and ELISA method were used to measure the concentration changes of five proteins which are related to inflammation and thrombosis processes.

The primary anti-inflammatory effects of NSAIDs is competitive inhibition of cyclooxygenases (COX) 1 and 2 which catalyze the rate-limiting conversion of arachidonic acid to the pro- and anti-inflammatory prostaglandins and thromboxanes. COX-1 and COX-2 convert arachidonic acid to prostaglandin H2 (PGH2), a committed step in prostanoid synthesis. COX-2 is responsible for production of inflammatory prostaglandins, which could be induced by inflammatory stimuli (33). COX-1 plays a major role in the generation of prostaglandins in gastric epithelial cells, such as prostaglandin E2 (PGE2), which plays an important role in cytoprotection (34). CRP displays several functions associated with host defense and be treated as a maker of inflammation (35). FII converts fibrinogen to fibrin and activates factors V, VII, VIII, XIII and functions in blood homeostasis, inflammation and wound healing (36). ALOX5 catalyzes the first step in leukotriene biosynthesis, and thereby plays a role in inflammatory processes. 

After strictly biological and toxicological research and test, pure CMC had been approved as food additive by (WHO) and (FAO). CMC has found wide applications in the pharmaceutical industry as a reliable carrier of drug (37, 38). In this study, the effect of CMC-Na was eliminated by administrating CMC-Na to control group. Therefore, it manifests that the effect of AEE is not related to CMC-Na. Tween-80 is widely used as emulsifier in food industry and drug production. In general, the body has a great tolerance to Tween-80 (39) but the biological potency of Tween-80 on target protein in this experiment is not investigated.

Compared with AEE groups, the ALOX5 values of integration of aspirin and eugenol group did not show statistical differences. This indicated that AEE may have similar effect to integration of aspirin and eugenol at molar ratio 1:1. The COX-1 and COX-2 values in AEE groups decreased with the increase of AEE dose. This change trend may result from the dose changes of salicylic acid and eugenol from enzymolysis of AEE which is crucial for inhibition of COX-1 and COX-2 (26). The COX-1 and COX-2 values in eugenol group were significantly lower than the values in CMC-Na group, which indicated that eugenol had influence in COX-1 and COX-2 and also showed positive impact on inflammation. However, the effect of aspirin on COX-1 and COX-2 was stronger than eugenol.

The CRP concentration of rats in low-, medium- and high-dose groups did not have great differences. However, its concentration was decreased compared to CMC-Na, eugenol, and aspirin groups. The reduction of CRP in AEE groups may be caused by synergistic effect of aspirin and eugenol. In addition, CRP concentration in the high-dose AEE group was higher than low- and medium-group, which may be caused by the side effects of salicylic acid from hydrolysis of AEE (26). The concentration change of FII in AEE groups appeared dose-dependent relationship and showed significant differences in comparison with CMC-Na and eugenol groups. The results in CMC-Na and eugenol groups were very close, which may indicate that eugenol have little or no effect on FII. There were statistical differences among aspirin, medium-dose AEE and integration of aspirin and eugenol three groups (same molar quantity). The differences in three groups may be from eugenol which increased the effect of aspirin on FII.

Like many other drugs, NSAIDs are associated with a broad spectrum of side effects, including gastrointestinal and cardiovascular events, renal toxicity, increased blood pressure, and deterioration of congestive heart failure. The side effects of AEE were not evaluated in this experiment. More studies are necessary to investigate on drug action of AEE such as side effects, aspirin-eugenol interactions, and clinical use.

From the results of the experiment, the concentrations of all key endogenic bioactive enzymes were reduced in AEE groups, which indicated that AEE had positive effects on anti-inflammation and anti-thrombosis. From the view of chemical-protein interactions, the results showed that AEE had stronger effects than aspirin and eugenol. Meanwhile, the effect difference between medium-dose and high-dose AEE groups was not significant. Therefore, it may be suggested that the clinical use dose of AEE is 100 mg/kg. In the present study, it is possible to conclude that AEE is a good candidate for the development of new anti-inflammatory NSAIDs. At the same time, these findings lay the groundwork for further pharmacological and clinical studies.

The study provides the first evidence that AEE is an effective COX inhibitor. AEE can significantly reduce the concentrations of CRP, ALOX5, COX-1, COX-2, and FII, which show better effects than that of aspirin and eugenol. Therefore, AEE could be a potential candidate for the treatment of inflammation as a new NSAID. Further studies of its clinical use and drug effects are necessary.
